# In defence of biodiversity

**DOI:** 10.1007/s10539-017-9587-x

**Published:** 2017-09-19

**Authors:** Joanna Burch-Brown, Alfred Archer

**Affiliations:** 1 Department of Philosophy, University of Bristol, Bristol, UK; 2 The Tilburg Center for Logic, Ethics and Philosophy of Science (TiLPS), Tilburg University, Tilburg, Netherlands

**Keywords:** Biodiversity, Conservation, Environmental ethics, Ecology, Philosophy of ecology, Eliminativism, Philosophy of biology

## Abstract

The concept of biodiversity has played a central role within conservation biology over the last thirty years. Precisely how it should be understood, however, is a matter of ongoing debate. In this paper we defend what we call a *classic multidimensional* conception of biodiversity. We begin by introducing two arguments for eliminating the concept of biodiversity from conservation biology, both of which have been put forward in a recent paper by Santana (Biol Philos 29:761–780. doi:10.1007/s10539-014-9426-2, 2014). The first argument is against the concept’s scientific usefulness. The other is against its value as a target of conservation. We show that neither of these objections is successful against the classic multidimensional conception of biodiversity. Biodiversity thus understood is important from a scientific perspective, because it plays important explanatory roles within contemporary ecology. Moreover, although it does not encompass all valuable features of the natural world, this does not show that we should abandon it as a target of conservation. Instead, biodiversity should be conceived as one of many grounds of value associated with ecosystems. This is consistent with concluding that a central aim of conservationists should be to protect biodiversity.

## Introduction

Should we protect biodiversity? Amongst conservationists, there is a widespread consensus that we should. In the Convention on Biological Diversity (1992), 193 UN signatories agreed ‘to achieve by 2010 a significant reduction of the current rate of biodiversity loss at the global, regional, and national level as a contribution to poverty alleviation and to the benefit of all life on Earth’ (UNEP 1992). A group of leading ecologists begin a recent paper in *Nature* with the thought that ‘The most unique feature of Earth is the existence of life, and the most extraordinary feature of life is its diversity’ (Cardinale et al. 2012). Biodiversity is often cited as an important consideration by both policy-makers and scientists, and concern for the loss of biodiversity is often seen as a central reason to protect ecosystems. For example, a study of land-use pressure in Southeast Asia has found that an extra 8.5 million hectares of rubber plantations will be required by 2024 in order to meet the growing demand for rubber, alone; the paper warned that this increase will bring about ‘catastrophic biodiversity impacts’ and ‘substantially exacerbate the extinction crisis in Southeast Asia’ (Warren-Thomas, et al. 2015, p. 7). In light of such concerns, 2010–2020 has been designated the ‘UN Decade of Biodiversity’.

However, it has been suggested that the concept of biodiversity is simply too inclusive and ambiguous to be useful (e.g. MacArthur 1972, p. 197). In a recent paper, Carlos Santana (2014) has taken this position, arguing that we should abandon the concept of biodiversity. He argues that it is problematic from a scientific point of view, on the grounds that it is too multi-dimensional to play a useful explanatory role in biological sciences. He also argues that biodiversity is a problematic focal point for conservationists on normative grounds. Preservation of biodiversity is often treated as the core aim of conservation, but many valuable features of the natural world are not reasonably viewed as aspects of *diversity*. Biodiversity does not capture all of what Santana calls ‘biological value’, and not all forms of diversity are valuable; thus the concept is both too broad and too narrow to be the primary target of conservation. Santana argues that we should focus directly on the features that are valuable in any given context, and do away with the concept of biodiversity.

This raises an important challenge for ecological science and environmental policy. If the challenge were successful then this would have important implications for ecological scientists and policy makers, as it would show that the current focus on biodiversity is misguided. This could provide reason to substantially change the goals of conservation science and policy.

In this paper we defend the importance of biodiversity. In the section entitled ‘What is biodiversity?’, we briefly introduce what we call the *classic multidimensional* conception of biodiversity. In ‘Two philosophical challenges for biodiversity’, we outline two challenges for philosophers seeking to defend biodiversity as an object of scientific interest and normative significance, by drawing on Santana (2014). We then consider two alternative conceptions of biodiversity developed by Sarkar (2005) and Maclaurin and Sterelny (2008), showing that although both are interesting, neither is fully satisfying. In ‘The conceptual importance of biodiversity’ we show that biodiversity in the classic multidimensional sense plays important explanatory roles in contemporary ecology, and thus is not vulnerable to the first, conceptual challenge. In the final section, we turn to the normative importance of biodiversity. We suggest that although biodiversity does not capture everything that is valuable about ecosystems, this need not undermine its normative importance. On our view, biodiversity is both instrumentally and non-instrumentally valuable, and should be conceived as one of many ‘grounds’ of value associated with ecosystems and the living world. We propose that this way of viewing biodiversity is both independently plausible and fits with one widespread conception in ecology.

## What is biodiversity?

According to what we call the *classic multidimensional* view, biodiversity refers to ‘the variety of life, in all of its many manifestations’ (Gaston 2011). On this conception, biodiversity can be broadly conceived as the variation or heterogeneity of living things, across all scales and levels of organization (Gaston 2011; Gaston and Spicer 2013; Spicer 2006). There are innumerable dimensions along which living things can be compared, so it is impossible to describe biodiversity as a simple magnitude (Gaston 2011). In assessing the diversity of two things, such as two habitat patches, we will always be comparing them with respect to certain dimensions and not others (Page 2010). To *fully* describe a region’s biodiversity would be impossible. Given limited resources, choices must be made regarding which aspects of diversity to focus on. Which dimensions we treat as most important will depend upon our explanatory purposes, our background theory (such as beliefs about which aspects of diversity might have a causal effect on the behaviour of a community) and our normative purposes (such as which dimensions we have particular reason to care about or value).

Classic multidimensionalists tend to reject the ambition of arriving at a small cluster of proxies for biodiversity, or describing biodiversity as a simple magnitude. Instead, they hold that *methodological pluralism* is necessitated by the variety of dimensions along which biota vary, and the variety of reasons we have for taking an interest in this diversity. Consider the example of a salt marsh. An ecologist studying the resilience of salt marshes to climate change might focus on the importance of ‘response’ diversity amongst marshland flora given changes to seasonal events, water temperatures and flow-rates. An evolutionary biologist might focus on processes of speciation amongst marsh-dwelling organisms. The department of commerce might be concerned with potential impact of declining diversity on the viability of commercial fisheries and tourism. A local naturalist group might take a particular interest in visually striking diversities of plant architecture or visible aquatic life. All of these groups are interested in specific components of the marsh’s biodiversity, and there is (according to the classic multidimensionalist) no simple cluster of measures that will serve all of their interests.

The classic multidimensional view is reflected in the work of many ecologists (e.g. Gaston 2011; Gaston and Spicer 2013). Attractively, it allows the study of biodiversity to fit neatly with research from diversity and complexity sciences more broadly (e.g. Page 2010). It also fits with the definition adopted in the 1992 UN Convention on Biological Diversity (CBD) (Article 2):

‘Biological diversity’ means the variability among living organisms from all sources including, *inter alia*, terrestrial, marine and other aquatic ecosystems and the ecological complexes of which they are part; this includes diversity within species, between species and of ecosystems.

The CBD definition draws attention to variation across all kinds of organisms, ecosystems, and scales. Although the definition is broad, it is also informative. It indicates that biological diversity specifically has to do with variability. Thus we suggest that the classic multidimensional view could be called a *variationist* conception of biodiversity. It is variationist because it places central emphasis on variability; describing biodiversity will require describing *heterogeneity* amongst organisms, communities and ecosystems.

Variationism about biodiversity also comes in more restrictive forms. For instance, some scientists hold that species are so important that they treat biodiversity as synonymous with species diversity.^1^ Others allow that biodiversity includes more than just species, but restrict it to *explanatorily significant* variation. For instance, Maclaurin and Sterelny (2008) see species richness as centrally important but also go beyond it to include morphological disparity and phenotypic variation. We see *classic multidimensionalism* as referring to the more inclusive view that biodiversity encompasses any and all variation amongst living things, regardless of whether this variation helps to explain other ecological or evolutionary outcomes. It is then up to scientists and others to enquire into which aspects of biodiversity are of interest from a scientific, intellectual, aesthetic, pragmatic or ethical point of view.

Classic multidimensionalism, then, is a variationist view, but not all ways of conceptualizing biodiversity give such an important role to *diversity* as such. Variationism is one of two dominant approaches to thinking about biodiversity. The other is what might be called an *inclusive normative* conception. On this view, ‘protecting biodiversity’ signifies something like protecting biotic communities, the biosphere, or even nature as a whole. As Bryan Norton puts it, biodiversity must ‘capture all that we mean by, and value in, nature’ (Norton 2006, p. 57). ‘Protecting biodiversity’, on this conception, is a matter of inclusively protecting any range of ecological features that are taken to be valuable. Many public uses of the term ‘biodiversity’ seem to assume an inclusive normative conception. For instance, the popular open source software called InVEST, which was developed with support from the World Bank, the Rockefeller Foundation, WWF, Nature Conservancy and Stanford University amongst others, is used by conservancy organizations and companies to assess ‘natural capital’, and distinguishes *biodiversity* from *ecosystem services*. It defines ecosystem services as benefits to human beings, and biodiversity as referring to living communities considered in their own right, apart from their usefulness to human beings. *Habitat quality* and change over time are used as proxies for biodiversity, and these are assessed by modelling Land Use and Land Cover (LULC), focusing on the ways in which human activities affect a given habitat patch. There is nothing about this approach that takes biodiversity to be a matter of *diversity* as such. Instead, on this assessment an area with high biodiversity will be an area of the biosphere that is comparatively uncompromised by human activity, whereas an area with low biodiversity will be one highly compromised by human activity.

Even in contexts where biodiversity is explicitly defined as the variety of life, it may be that the implicit conception of biodiversity at work is much broader. For instance, signatories to the UN Convention on Biological Diversity promised to protect biodiversity as a contribution ‘to poverty alleviation and to the benefit of all life on Earth’ (UNEP 1992). There is good reason to think that the aims of alleviating poverty and benefiting life on Earth depend as much upon preservation of biological abundance, ecological complexity and specific ecological structure and function (such as pollination), as they do on the *variety* of life. Thus although the explicit CBD definition focuses on variation, the purposes of the convention seem to imply a wider conception of biodiversity. Likewise, popular ideas about biodiversity often seem to be normatively rich and inclusive. For instance, research into public attitudes in Scotland found that regardless of scientific knowledge, members of the public had rich mental constructs of biodiversity, associating it (although not uncritically) with ideas of harmony and balance in human relationships to nature, and the interconnectedness of living things (Fischer and Young 2007). By contrast, a ‘variationist’ view like classic multidimensionalism better reflects a technical conception often adopted in fields like ecology.

It is not our aim to deny the potential value of alternative conceptions; on the contrary, there may be a range of useful ways to conceptualize biodiversity. If other conceptions are also retained, then important work remains to be done in the future to clarify the relationships between them. However, in this paper, we concentrate on the classic multidimensional conception, according to which *x* contributes to biodiversity if and only if it contributes to variation or heterogeneity amongst living things, considered across all scales and levels of organization. Our first aim is to show that biodiversity understood as ‘the sheer variety of life’ plays two important roles within biological sciences—as a phenomenon to be described and explained, and as a higher-level feature of the biological world that may potentially help to explain other phenomena. Our second aim is to show there are good *pro tanto* reasons to think that biodiversity thus understood is something to be valued and protected. The sheer variety of life is seen by many people as a *wondrous* feature of the natural world. In adopting this perspective, people seem to value biodiversity in general and not merely diversity along specific dimensions. This suggests that biodiversity in the classic multidimensional sense has an important role to play in both science and environmental ethics.

## Two philosophical challenges for biodiversity

Santana (2014) has argued that although the concept of biodiversity has been scientifically and politically influential, it faces fundamental philosophical problems. In light of these problems he defends an eliminativist view, arguing that we should do away with the concept of biodiversity altogether. Santana’s objection to the concept of biodiversity has two parts.

His first objection is on conceptual, scientific grounds, and is that the concept is multi-dimensional in a way that limits its usefulness. As noted above, it is widely recognized that there are many different components of biological diversity. In considering the heterogeneity or homogeneity of an ecosystem, we may be interested in any number of features, including genetic and phenotypic variation; diversity between species; diversity with respect to functional roles in the ecosystem (e.g. primary producers, herbivores, carnivores; canopy dwellers, ground feeders etc.); and variation in the composition or network structures of whole communities (Page 2010). Diversity, then, can be measured along innumerable different dimensions. Moreover, these different dimensions may vary independently of one another. A community might have many species, but low genetic variation. It might have many species in small numbers, but be dominated by just a few, and thus be relatively homogeneous in behaviour and structure. Given that there are many dimensions by which ecosystems can be compared, and along which they may be more or less heterogeneous, no single measure is likely to capture the overall diversity of a community. Biodiversity is a fundamentally multidimensional concept (Purvis and Hector 2000).

Santana argues that if the different dimensions of biodiversity do not reliably vary with one another, then we are better off appealing just to the specific aspect of diversity that is relevant for a given explanatory purpose, and doing away with the overarching concept. ‘Against pluralists, who hold that biodiversity consists of distinct but correlated properties of natural systems, I argue that the supposed correlations between these properties are not tight enough to warrant treating and measuring them as a bundle’ (2014, p. 761). The overarching concept is redundant, he thinks, if each time the term is used, the speaker in fact mean something more specific, the content of which is evident from explanatory context; and if there is no way to measure biodiversity as a whole. In one context, the important feature may be herbivore diversity; in another it might be variation in lifecycles amongst grassland insects. If the many dimensions of interest are distinct and vary independently, then little may be gained by employing the concept of biodiversity. Instead the focus in each case should be on whichever dimensions are explanatorily relevant. That is Santana’s conceptual objection to biodiversity.

Santana’s second objection is on normative grounds. Santana observes that biodiversity is often used in practice as a catchall for the value of living organisms in natural environments. As Maier (2012) puts it, many conservationists subscribe to the ‘biodiversity project’, according to which biodiversity is meant to capture the core of what is valuable about the natural world. However, this inclusive use of the term is problematic. For reasons that have been explored thoughtfully by others (e.g. Rawles 2004; Maier 2012) much of what we value about the natural world and living communities is not well-captured either by the broad concept of diversity, or by any of the individual measures commonly used to represent it. Santana’s conclusion is that conservation biologists should not seek to preserve biodiversity, which he suggests is an unhelpful placeholder. Instead, conservationists should aim directly for preserving what he calls ‘biological value’. Santana does not define biological value, but seems to mean something like the value associated with the biosphere, ecosystems, or communities of living things.

It may be helpful to position Santana’s argument in relation to wider debates regarding eliminativism and reduction in philosophy of science and ethics.^2^ Many philosophers of science have held that scientific progress takes place in part by replacing general concepts with more precise ones (Kemeny and Oppenheim 1956). Replacing concepts might involve either eliminating the higher-level concept altogether (see e.g. Churchland 1981; Machery 2009); or breaking it down into its component parts and explaining the higher-level concept in terms of a reducing base. Arguments for eliminating a concept from a theory often hinge on the question of whether the concept picks out a natural kind. If it is shown that there is no natural kind to which the concept refers, then there might be reason to eliminate the concept from the theory. One way of showing that there is a natural kind is to show that observed correlations between properties (such as observations of teeth, claws and manes) can be ‘projected’ to other instances, allowing for generalized claims about the kind (such as the claim that lions have manes) (Goodman 1954). Thus one way of arguing against the existence of a natural kind is to deny the existence of some set of properties shared by the members of the kind. For instance, Edouard Machery (2009) argues that the phenomena referred to by the term ‘concept’ are heterogeneous, with no core set of properties shared by the important instances of the term, and concludes that ‘concept’ does not refer to a natural kind. Similarly Griffiths argues that emotions are highly heterogeneous (1997, 2004) so that the kinds of properties that are centrally important to some kinds of emotion are absent in others, leaving only a trivial set of shared characteristics. Santana adopts this kind of strategy, arguing that we should eliminate ‘biodiversity’ on the grounds that the various properties supposedly associated with biodiversity (species richness, disparity, genetic diversity and so on) do not always rise and fall together. An ecosystem might be diverse in one respect and not in another. He concludes that we should describe diversity along specific dimensions, but do away with the umbrella concept of biodiversity.

One response would be to claim that this argument assumes too strong a standard for biological kinds. For instance, on the Homeostatic Cluster Properties conception of natural kinds (Boyd 1991), there may be *no* properties that all instances of a kind share, but if the important properties tend on the whole to cluster together, and if a mechanistic explanation can be given for this clustering (such as species boundaries), then there might be a biological kind. One strategy for replying to Santana, therefore, would be to seek to show that the dimensions of diversity do tend to be reasonably strongly clustered, and that there are underlying mechanisms for this clustering. Theoretically it might be possible to show that systems with high diversity of species tend to also have higher phenotypic diversity, genetic diversity, and morphological diversity (colours, shapes, sizes, behaviours, body plans and so on) than systems with low diversity of species. Even though these dimensions are not tightly correlated and can come apart (so that a system might have high species diversity but low morphological diversity, for instance), they might nevertheless tend to be sufficiently correlated for biodiversity to qualify under the Homeostatic Cluster Property conception of biological kinds. A different strategy might be to emphasize that the property of biodiversity is a natural kind that can be multiply realized. It does not matter that the associated properties do not tightly covary; different biota can be biodiverse in different ways, while sharing a common property of high heterogeneity. The challenge for this strategy is to show that systems that are biodiverse in different ways really do share something important in common which means that the multiply-realizable property of diversity is a natural kind and not just a conventional description.

However, even if a concept does not refer to a natural kind, it might earn a place in scientific theory by being functionally useful in helping to systematize understanding (e.g. Boyd 1999). A functionally useful concept might be one that reveals the unity in apparently diverse phenomena. Showing that a concept increases understanding is less demanding than showing that it refers to a natural kind. For instance, even if on some views fragility is not a natural kind because it can be realised in such heterogeneous ways (with brittle paper being fragile in a different way from a glass), it might be a useful term in scientific explanation, because it reveals what various phenomena share in common (a disposition to break under stress) and thus helps to systematize understanding (see e.g. Kemeny and Oppenheim (1956) and subsequent literature). Since Santana claims that the concept of biodiversity serves no useful function, to answer his argument it is enough to answer this functional claim and show that biodiversity, understood as ‘the variety of life’, does have useful roles to play in systematizing understanding.^3^

## Two alternatives

Santana presents his eliminativist view as an alternative to two other accounts of biodiversity that have attracted attention amongst philosophers. The first is Sahotra Sarkar’s ‘deflationary’ account (2005), and the second is a moderate multidimensionalism developed by Maclaurin and Sterelny (2008). We will present both views, and explain why we think that neither offers a fully satisfying alternative, although both are interesting.

### Sarkar’s account

Sahotra Sarkar defends an inclusive normative conception of biodiversity. At his most pragmatic, Sarkar proposes that biodiversity simply names ‘what is being conserved by the practice of conservation biology’ (2002, p. 132). He later adopts a less deflationary account, but still maintains that there should be a close link between the scientific concept of biodiversity and the normative aims of conservation. As he puts it, ‘The rationale for the creation of conservation biology as a discipline was the protection of biodiversity’ and ‘this normative goal—conservation—severely constrains how biodiversity should be conceptualized’ (2010, p. 131). A potential advantage of Sarkar’s approach is political. Protecting biodiversity is a popular goal around which environmental advocates have been able to organise support. Sarkar hopes that defining biodiversity with our normative purposes in mind will mean that it can continue to provide a useful basis from which to make conservation decisions.

However, as it stands Sarkar’s proposal also has potential disadvantages. First, the deflationary definition in itself does not easily ground an account of why biodiversity should be valued. Contrary to what Sarkar seems to suggest, protecting biodiversity cannot provide the *rationale* for conservation biology if biodiversity is defined as whatever the discipline seeks to protect. A rationale would need to provide some further, independent justification. This might include a descriptive component characterising certain features of ecosystems, and a normative component explaining why these features are valuable. One rationale that can be appealed to, for instance, is a sense of wonder at the fact that life has evolved such an extraordinary variety of forms (Wilson 1984). We can also appeal to ethical values grounded in this aesthetic, intellectual and spiritual appreciation, such as a moral conviction that the sheer variety of life should be valued and protected for future generations. The sense of wonder and awe can offer a rationale for conservation in a way that a purely deflationary account does not.

However, perhaps we can defend Sarkar’s view. Conservationists are often involved in uniquely attentive reflection on values associated with the natural world, and have provided rationales for their judgments. If conservation biology is one discipline where reflection about the natural world has been pursued most carefully, perhaps it will be fruitful to define biodiversity by looking towards conservation practice, supported by the rationales given by conservationists. Nevertheless, there remain objections to this approach. Conservationists are not the only people who have developed considered views on the value of the natural world. It might be problematic to define biodiversity by privileging the views of this particular community over others. Moreover, the current version of conservation practice may be an imperfect reflection of core conservation values. Another concern is that tying an empirical definition to normative values makes it difficult to have a transparent debate about which aspects of diversity are valuable, or about their importance compared with other practical values. The fact that there is likely to be reasonable disagreement over which aspects of diversity are valuable may give us reason to maintain greater independence between the empirical concept and the normative values related to it.

A final worry is that Sarkar’s conception of biodiversity seems to assume that conservation biology is the primary context in which biodiversity is important. In fact the concept of biodiversity plays a range of other important roles in the biological sciences. For instance, many ecologists study ‘biodiversity effects’, seeking to discover to what extent, if any, the overall heterogeneity of an ecosystem helps to explain its behaviour. It could be problematic to define biodiversity as whatever conservation biologists seek to conserve, if this is not the conception underlying many empirical and theoretical uses of biodiversity within biological sciences.

Given that the term is often used in ecology to denote *heterogeneity*, taking biodiversity as the governing concept of conservation could potentially lead conservationists to focus too much on features related to *diversity* and too little on other important characteristics. The diversity of life is of great conservation interest, but so are many other features of ecosystems, such as network structure, habitat structure, community complexity, abundance, and so on. It might be better to develop a more *articulated and differentiated* range of conservation concepts. Developing a wider range of popular conservation concepts might lead to richer and more nuanced understandings of ideal conservation aims.

### Maclaurin and Sterelny’s account

A second alternative to eliminativism can be found in an account developed by Maclaurin and Sterelny (2008).^4^ Like Sarkar, Maclaurin and Sterenly are motivated by a specific decision-making problem from conservation biology—namely, the problem of ‘place prioritization’, or choosing which habitats to conserve, given scarce resources. Given this practical aim, they hope to arrive at a general measure of biodiversity that is both theoretically motivated and empirically tractable. The general measure of biodiversity, they say, should allow conservationists to objectively rank the diversity of different biota. Their hope is to identify a tractable, general-purpose measure, which captures the most explanatory dimensions of diversity. Maclaurin and Sterelny defend the view that species richness is centrally important, but allow that other dimensions such as morphological disparity and phenotypic variation may sometimes need to be measured separately.

Maclaurin and Sterelny start their account by observing that in practice scientists often either count or else catalogue species as an operational measure of biodiversity. They seek to provide theoretical support for this approach, by arguing that the number of species is an objectively central dimension of biodiversity. They state that evolutionary species or ‘the collection of independently evolving lineages in a region’ is ‘a key component, perhaps the key component, of that region’s biological diversity’ (2008, p. 40). Species diversity is generally a more central dimension of biodiversity than variation within (macroscopic) species, they argue, because variants within species will tend to regress to the mean over time, through sexual recombination of genes. By contrast, differences between (macroscopic) species are more stable (Eldredge 1995). Thus they state that ‘There is an important difference, on this picture, between a single widespread and phenotypically variable species (like the common brushtail) and a set of closely related species’ (40). The latter set of phenotypes, they say, will be ‘entrenched by speciation mechanisms, and hence will survive minor ecological changes … the other set is much more fragile in the face of relatively minor ecological change’ (40). Since species boundaries preserve variation, they say, a list of species provides ‘a catalogue of phenotypic variety and of the potential evolutionary resources available’ in a region (p. 40). Moreover, they say, an evolutionarily-informed catalogue of species can serve as a good proxy for other dimensions of biodiversity much of the time (e.g. pp. 7; 25). This is in part because species often structure other aspects of diversity. For instance, gathering information on species composition is often a good basis from which to recover information about functional or trait diversity.

However, they acknowledge that simply *counting* species may not provide enough information about the diversity of a biota for many conservation purposes. Virtually all biologists agree that there is more to biodiversity than species diversity, and more to species diversity than so-called ‘species richness’ (ecologists’ term for the *number* of species). For instance, species richness provides no information about either the distinct characteristics of those species, nor the *relative abundance* of the species present. A community composed of three species of maple is as species-rich as a community composed of one species of maple, one species of pine, and one species of frog, because both have three species—but the latter is more diverse than the former, because it has greater *disparity*. The organisms in the second group are substantially more different from one another than are the organisms in the first group. Moreover, two groups might have the same number of species, but if one ecosystem is overwhelmingly dominated by just a couple of species while the other has numerous species in great abundance, the latter seems intuitively to be more diverse. Assessing diversity is therefore not only a matter of counting species.

Thus Maclaurin and Sterelny go on to ask what, if anything, should be added to supplement species-counts in order to arrive at an adequate description of biodiversity for conservation decision-making. They ask whether species richness is a good guide to *phenotypic* diversity (chapter 3–4) and whether species richness is a good indicator of the developmental resources that might generate future diversity (chapter 5). They also consider attempts to reflect *disparity* (the extent of the morphological differences between organisms) by mapping organism geographies in ‘morphospace’ (chapter 4). Finally, they consider the proposal that *ecosystem diversity* might be a distinct dimension of diversity. In each case, they demonstrate that species richness is not always an adequate measure, but conclude by reaffirming the general utility of counting species.^5^

Maclaurin and Sterelny explore numerous valuable lines of enquiry in their account. However, many ecologists would object to placing as much emphasis as Maclaurin and Sterelny do on species richness in the assessment of biological diversity. Their account tends on the whole to downplay the distinctive importance of other forms of variation. For instance, their argument for the primacy of species seems to downplay the distinctive importance of other forms of diversity, such as intra-species variation. Within-species variation has different causal effects than between-species diversity, at least for macroscopic species, because it allows for exchange and recombination of genetic information, whereas exchange of genetic information between macroscopic species is impossible. Homogeneity *within* a species increases its vulnerability to environmental threats, predators, and pathogens, because it leads to inbreeding and thus greater expression of deleterious recessive genes. For example, consider the importance of within-species variation for adapting to climate change. It is thought that climate change will lead to shifts in the timing of flowering and other lifecycle events for plants. It is predicted that these shifts may mean that plant flowering fails to synchronize with pollinator flight activity, leading to lack of pollination for the plants and lack of food for pollinators—and thus resulting in local extinctions (Memmott et al. 2007). Diversity in the timing of lifecycle events within species might give specialist plant and pollinator populations the capacity to adapt to climate change, whereas lack of within-species variation will make specialist populations vulnerable to extinction. Thus intraspecific variation is an important form of diversity that is not revealed by counting species. It could be replied that counting species nevertheless serves as an adequate proxy for within-species variation, on the grounds that ecosystems with greater species diversity are likely to also have higher diversity within each population. However, many habitats with a large number of unique and distinctive species (such as islands) have small populations, low genetic diversity within these populations, and a high vulnerability to extinction. Counting species does not provide a good indicator of the long-term trajectory of diversity in these ecosystems.

Within-species variation is just one of many aspects of biological diversity not captured by species measures. As another example, *landscape ecologists* study the ways in which diversity in habitat arrangements shape ecological outcomes. They argue that variety in spatial distribution and patterning of habitat is a centrally important aspect of biodiversity. Differences in spatial distribution and habitat patterning can have both ecological and evolutionary implications. Information about habitat patterning and spatial distribution cannot be recovered by listing species. Similarly, it has been argued that evolutionary lineage is less important than *functional diversity* (diversity of ecological roles) in explaining ‘biodiversity effects’ on ecosystem behaviour (e.g. Naeem 2012, p. 35. See also Petchey and Gaston 2006; Schleuter et al. 2010). Related points can be made about the limitations of species measures for capturing relevant diversity of other kinds, such as response diversity (diversity of response to environmental change), life history and trait diversity, beta-diversity (between-patch comparisons of community composition), network diversity (diversity in the structure of interaction networks, for instance amongst pollinators and plants) and so on. Maclaurin and Sterelny’s account gives less attention to these important aspects of diversity than might be warranted by current ecological science. In our view, MacLaurin and Sterelny overemphasise the importance of species diversity (and species richness in particular) relative to other aspects of diversity.

A second objection is that Maclaurin and Sterelny take ‘place-prioritization’ as the paramount problem facing conservationists, and go on to define biodiversity in relation to this problem. However, conservation is not reducible to the problem of selecting which places to preserve. Much conservation work centres on other questions, related to managing or restoring habitats so as to protect ecosystem structure and function. Many of these questions require a descriptive and mechanistic understanding of biodiversity, and not simply an assessment of the *quantity* of biodiversity. For instance, traditional grazing in the Netherlands resulted in a great diversity of plants, and correspondingly great diversity of arthropods, such as beetles (Poschlod and Wallis de Vries 2002). By contrast, modern land use in these areas has resulted in homogeneity of plant structure and reduced diversity of arthropods. Returning to traditional grazing is expensive, and has not always resulted in recovery of plant and arthropod diversity (Poschlod and Wallis de Vries 2002). It has been argued that improving restoration requires a mechanistic understanding of grassland biodiversity, such as an understanding of the diversity of reproductive, developmental, dispersal, and synchronization strategies amongst organisms in a target community (van Noordwijk et al. 2012; van Noordwijk 2014). In this context, it is less important for analyses of biodiversity to provide simple rankings between habitats, and more important for them to contain substantive information to guide conservation strategies (van Noordwijk et al. 2012; van Noordwijk 2014). Thus many ecologists would object to using the ‘place-prioritization’ problem as the basis from which to generate a *general* definition and measure of biodiversity. Instead, there are advantages to defining the umbrella concept of biodiversity more broadly, and then examining specific dimensions of it.

A final difference between Maclaurin and Sterelny’s view and ours is that they associate biodiversity with *explanatory* diversity, whereas on our view, the umbrella concept of ‘biodiversity’ includes any and all variation amongst biota. Maclaurin and Sterelny hone in on explanatory diversity because they hope to arrive at a measure that shows biodiversity to be instrumentally important for human concerns, most notably for the stable provision of ecosystem services (119–123; chapter 8). By contrast, we adopt a more inclusive conception of biodiversity, and hold that it is a further question which aspects of biodiversity turn out to be important and for what purposes.^6^

Is it better to define biodiversity restrictively as explanatory variation, or inclusively as any and all variety amongst living things? The more restrictive definition may seem at first sight to be more tractable, but it runs counter to scientific practice in some important ways. In ecology it is generally an open question whether and to what extent biodiversity is explanatory of other ecological outcomes. As the examples above have illustrated, scientists often operationalize biodiversity to focus on features of diversity that they expect might be explanatorily important, but in doing so they are not attempting to find out whether these features really are components of biodiversity. Instead, they are attempting to see which components of biodiversity have explanatory importance. That seems to speak in favour of the more inclusive, classic multidimensional conception. On the *explanatory diversity* view, we wouldn’t know whether a given kind of variation was part of biodiversity until we knew that it was causally important. However, given the possibility that diversity might be causally important in some contexts but not others, that seems to introduce the complication that what counts as biodiversity might vary from system to system. It seems more straightforward to say that all aspects of life’s variety are part of biodiversity, but that some aspects of this diversity have greater causal or explanatory importance than others. That leaves scientists free to examine the hypothesis that biodiversity or some component of it has explanatory value, without committing to that view from the start—i.e. to treat it as a potential explanans.

## The conceptual importance of biodiversity

Given the multi-dimensionalism and methodological pluralism we have defended, it might seem that we should follow Santana and concur that the higher-level concept of biodiversity is redundant. Even if the *best* interpretation of biodiversity is as ‘the variety of life’, that does not mean the concept will necessarily turn out to be useful, either for scientific theory or for environmental ethics. If the concept had no explanatory or normative importance, then we might be able to do away with it. For instance, if it were true that whenever scientists referred to biodiversity, they were in fact referring to some more specific component of it, and this could be seen from explanatory context, then there might be a case for thinking that the umbrella concept of biodiversity was playing no role. By analogy, one might attempt to argue that ‘size’ as such plays no explanatory role in science, because each time someone refers to size they in fact mean some specific dimension such as volume, mass, height, etc. However, we shall now argue that the umbrella concept of biodiversity as ‘the variety of life’ does have important roles to play in both scientific explanation and in environmental ethics.

### Biodiversity as explanandum

First, biodiversity has an important role to play in science as an explanandum—which is to say that scientists often treat biodiversity as an aspect of living systems to be descriptively characterized and explained. The sheer variety of life is identified by many biologists as a central object of scientific interest. For example, in 1908, at a Linnean Society event marking the fiftieth anniversary of the readings of the Darwin-Wallace papers, the following comments were offered by Alfred Russel Wallace:

Why did so many of the greatest intellects fail, while Darwin and myself hit upon a solution to this problem?… First (and most important, as I believe), in early life both Darwin and myself became ardent beetle-hunters. Now there is certainly no group of organisms that so impresses the collector by the *almost infinite number of its specific forms, the endless modifications of structure, shape, colour, and surface*-*markings that distinguish them from each other, and their innumerable adaptations to diverse environments…* Again, both Darwin and myself had, what he terms ‘the mere passion of collecting,’—not that of studying the minutiae of structure, either internal or external. I should describe it rather as *an intense interest in the mere variety of living things*—the variety that catches they eye of the observer even among those which are very much alike, but which are soon found to differ in several distinct characters…

*It is the constant search for and detection of these often unexpected differences between very similar creatures, that gives such an intellectual charm and fascination to the mere collection of these insects;* and when, as in the case of Darwin and myself, the collectors were of a speculative turn of mind, they were constantly led to think upon the ‘why’ and the ‘how’ of all this wonderful variety in nature—this overwhelming, and, at first sight, purposeless wealth of specific forms among the very humblest forms of life (quoted in Berry 2008, italics added).

For Wallace, the central object of scientific interest was the variety of life as such. His focus cannot be captured in any particular component such as species richness, nor certainly would he recognise the idea of biodiversity as reducible to a simple magnitude. Instead, he sought to characterise and explain the existence of innumerable forms, differing in innumerable ways, as well as the underlying processes by which these ‘variations on themes’ come to exist. This interest in variation reflects a much longer tradition of research by naturalists. Early taxonomists focused on cataloguing intra- and inter-specific diversity; while naturalists in the nineteenth century began moving beyond taxonomical questions to seek unifying theories to explain the distribution and abundance of living things, and the evolutionary processes underlying biological variety. Contemporary research extends this interest to the microscopic levels of genetic variation, and to higher-level variation in community structure and across landscapes.

It is worth noting that in this role, as object of scientific study to be characterised and explained, biodiversity is not simply a magnitude. Scientists and naturalists are interested not just in the amount of diversity, but in characterising the qualitative details of that diversity—the specific differences and similarities between organisms, communities, landscapes and ecosystems. Amongst the central aims of biology, then (and particularly of branches like ecology, biogeography and conservation sciences) is the characterisation and explanation of biological diversity—what it is like and how it comes to exist.

One might object that the view of biodiversity as the variety of life is too broad. It might seem to make the study of biodiversity synonymous with the study of biology. However, this is not right. Biodiversity refers to biological heterogeneity or *diversity*. Diversity is a fundamentally relational concept (Page 2010), because it is a matter of the similarities and differences between two or more objects. Biology as a science encompasses many questions that are not fundamentally relational in this way, and are instead concerned with the close understanding of individual biological forms. For example, many parts of biology essentially focus on explaining how things work, such as how cells are replicated, how energy is produced, how genes function at a molecular level, and so on. It may be part of these studies to look at biological diversity (diversity in forms of cells, or forms of energy production, for instance) but the study of variation does not encompass the whole of these sciences. Biodiversity does not include ‘all of biology’, although the study of variety is clearly of central interest within biological science.

For similar reasons, one might worry that defining biodiversity as the variety of life could lead to the view that protecting biodiversity requires protecting every living thing. For instance, Sarkar has suggested that if biodiversity is conceived as ‘the variety of life, in all of its many manifestations’ (Gaston 2011), then biodiversity simply becomes equivalent to ‘all of biology’, and the preservation of biodiversity on this definition would require the preservation of all living organisms (Sarkar 2005, 2010). He writes that ‘If biodiversity is taken to be all of natural variety at every level of taxonomic, structural, and functional organization, the concept cannot be operationalized for conservation in practice: the goal of conservation would become all biological entities’ (Sarkar 2010). Similarly, Wilson writes that ‘Biologists are inclined to agree that it is, in one sense, everything’ (1996, p. 1); and Yrjö Haila worries that ‘if an issue covers “everything” then how can it simultaneously acquire analytic clarity and strength?’ (2004, p. 55) and ‘how do you stabilize research on “everything”?’ (2004, p. 58). Santana often returns to this worry, objecting that ‘we cannot save all of biology’ and thus that a narrower definition is required (Santana 2014, pp. 763, 765, 772, 777). However, again this is not quite right. Biodiversity refers to biological *heterogeneity*, and preserving heterogeneity does not depend on preserving all living things. There are many conservation choices that clearly preserve greater heterogeneity than others. For instance, conserving healthy habitat within the ‘biodiversity hotspot’ regions such as South Africa, Madagascar, Ecuador or South East Asia, will, on almost any reasonable composite measure, conserve more biodiversity than saving a comparably sized area of forest in Ohio (although we have good reason to protect these areas of natural beauty as well).

Finally, it might be objected against our view that although naturalists like Wallace often appeal to an interest in the sheer variety of life, in fact we are attracted to coral reefs and rainforests because of charismatic organisms—the bright colours and mating dances of birds of paradise, for instance, or the exotic anatomy of longihorn beetles. It is not *diversity* as such but the ‘identities’ of charismatic organisms that attract scientific interest. Maier (2012) makes this argument. In response, we agree that charismatic animals like predatory cats, pandas, and butterflies often stimulate efforts to protect the natural world. However, valuing particular organisms is compatible with also valuing biodiversity itself, as an important source of interest in the living world. Moreover, our interest in the identities of particular organisms is itself often connected to an interest in the extraordinary variety of life. The coral reef is interesting in part because it has such a complex community structure, composed of organisms with such widely varied characteristics—sea cucumbers, cephalopods, coral polyps, moray eels, fans, puffer fish, grouper and so on. It is not only the charismatic identities of individual organisms, nor any particular dimension of diversity, but in many cases the sheer variety across innumerable dimensions that makes certain ecologies like coral reefs objects of unique scientific interest.

### Biodiversity as explanans

The arguments we have given so far are adequate to secure a valuable place for the concept of biodiversity in biological sciences. However, biodiversity also plays a second role in contemporary ecology, which is as a potential explanans—that is, as a property that may partially explain an explanandum, such as some aspect of the behaviour of a community. Appeal to a higher-level property like diversity might increase the understanding of ecosystem behaviour by revealing the unity of apparently diverse phenomena. If heterogeneity is commonly associated with certain effects in complex systems, then knowing that a system has a high level of heterogeneity may help scientists to understand its behaviour.^7^

Scientists across many fields have explored the possibility that diversity is associated with characteristic patterns of behaviour in complex systems. Over a number of decades, ecologists have debated possible relationships between diversity and features like ecosystem stability or robustness, often arriving at competing conclusions (deLaplante and Picasso 2011; Justus 2011). In an attempt to clarify the message for the public, leading ecologists since 2005 have published a number of ‘consensus statements’ summarizing current agreement regarding relationships between biodiversity and ecosystem behaviour (Balvanera et al. 2006; Cardinale et al. 2012; Hooper et al. 2005; see also deLaplante and Picasso 2011).

According to the most recent such statement, seventeen ecologists write in *Nature* that ‘There is now unequivocal evidence that biodiversity loss reduces the efficiency by which ecological communities capture biologically essential resources, produce biomass, decompose and recycle biologically essential nutrients’ (Cardinale et al. 2012, p. 60). They also report agreement that current evidence supports the hypothesis that genetic, species and functional diversity tend to be associated with ‘insurance effects’, and that on average there is ‘greater temporal stability of a community property like total biomass at higher levels of diversity’ (2012, p. 60). They find that initial losses of biodiversity are generally associated with small declines in functioning, whereas increasing losses lead to accelerating impacts on ecosystem dynamics. Emphasising that their study has compared findings concerning genetic, species and functional measures of diversity, they argue that findings support the view that ‘there are general underlying principles that dictate how the organization of communities influences the functioning of ecosystems’ (2012, p. 60).^8^ In other words, they attempt to argue that it is not just diversity in any given dimension but also *overall* heterogeneity that is of ecological importance.

Many questions might be raised concerning the significance of these claims. For instance, one question regards the generalizability of findings. Do experiments thus far, many of which have been short-term and have focused on plant communities, have validity in more complex and dynamic ecosystems, and amongst less easily observed organisms? Another question concerns the problem of hidden variables. Is it possible that measures of diversity are tapping other, unobserved variable(s)? Can experiments thus far demonstrate that the property of heterogeneity is genuinely explanatory, or might outcomes be driven by other properties, such as identities of keystone species? Given the complexity of ecological processes, it is likely that such inferences will be subject to continued scrutiny, and that understanding will continue to evolve significantly from its current state. Nevertheless, for our argument it is not necessary to determine which effects exist, or how strong each effect is. It is only necessary to show that biodiversity in the classic multidimensional sense is serving as a potential explanans within ecology, with some justification. Given the evidence above, this seems to be true.

We are now in a position to return to Santana’s eliminativist argument. Santana interprets Maclaurin and Sterelny as implying that on a pluralist view, ‘biodiversity consists of distinct but correlated properties of natural systems’ (2014, p. 761). He draws the conclusion that the umbrella concept can only be useful if the component dimensions of diversity reliably co-vary. He then seeks to show ‘that the supposed correlations between these properties are not tight enough to warrant treating and measuring them as a bundle,’ (2014, p. 761). For instance species richness and relative abundance may vary independently of one another, and independently from other aspects of diversity, like phenotypic variation.

However, the pluralist or multidimensionalist view does not depend on the dimensions of diversity correlating with one another. As the above discussion shows, there are other conditions under which a higher-level concept like biodiversity is valuable. One possibility is that a higher-level concept might be useful because it ‘summarizes’ the interaction of many underlying mediating variables, whose relationships would otherwise be too complex to capture easily. Rather than appealing to each underlying variable and attempting to explain in any given context its relation to all of the others, it may be more explanatory to refer to the higher-level property—in this case, the property of diversity. The higher-level concept may thereby allow us to unify and explain what is shared in common across the various individual cases.

The higher-level concept of biodiversity may also be useful if the property of diversity tends to be associated with characteristic effects within whichever dimension it appears, so that (for instance) species diversity tends to have characteristic effects with respect to species, intraspecific diversity has the same kinds of effects within populations, and functional diversity has the same characteristic effects across functional groups. If this is true, then knowing that a community is highly heterogeneous overall could give us reason to expect certain patterns in community processes. Given the attraction of the broad explanatory power that might follow, it is likely that ecologists will continue to explore the possibility that there could be broad biodiversity effects, and thus to treat biological diversity as a potential or partial explanans with respect to ecosystem processes.

Even if the idea of biodiversity effects is rejected, however, there is still good reason to retain the concept of biodiversity within science. The term names a broad phenomenon of scientific interest—the variety of life—which may be too complex to be studied as a whole but the knowledge of which can be developed through the study of its parts. By analogy, ‘biology’ is a useful concept, as the study of living things, even though we can only approach this by studying component parts of the subject at a time. Scientists may see species diversity, functional diversity, and phenotypic variation as all being aspects of biological diversity, so that studying each of these contributes to understanding of the larger phenomenon of the variety of life. Given the important explanatory roles that biodiversity plays in contemporary ecology—as both explanandum and explanans—there is good reason at present for scientists to retain the concept.

## The normative importance of biodiversity

Those who accept everything that we have argued so far can agree that biodiversity has a useful role to play in ecological sciences. However, our argument up to this point does not show that conservationists should aim to preserve biodiversity. We might agree that biodiversity has a useful explanatory role to play in science without concluding that we should seek to protect it.

The normative role of biodiversity in ecological science has been the subject of an important challenge. Santana argues that the concept of biodiversity is problematic on normative grounds because it is often used as a catchall for everything that is valued about ecosystems or communities of living things. He notes that much of what we might reasonably value about living communities is not plausibly conceived of as diversity, and some forms of biological diversity are not valuable. He concludes that scientists, policy-makers and conservationists should avoid the concept of biodiversity and instead focus directly on whatever features are either empirically or normatively significant in a given context. Maier (2012) develops a similar argument. Unlike Santana, Maier accepts the conceptual coherence of biodiversity, but he argues that there is no reason to think that it is valuable, and thus argues that we should reject what he calls ‘the biodiversity project’.

In this section we will defend the normative role of biodiversity against this important challenge, focusing on Santana’s version of the argument, and showing that his argument does not support his conclusions. It is possible to agree with Santana that diversity does not capture everything that valuable about the living world, while still holding that the diversity of life is valuable in and of itself, and worth protecting. The variety of life is one of many features to be valued in the natural world, and conservationists should aim to protect it.

### Limitations of biodiversity

Santana claims that much of what we value in the natural world is not best conceptualized in terms of diversity (2014, p. 773). This is undoubtedly correct, and we accept this aspect of Santana’s argument. Framing conservation concerns primarily in terms of diversity could potentially lead to neglecting other components of ecological value. For instance, it might make it hard to articulate the importance of protecting relatively undiverse communities such as many woodlands, which may nevertheless be ecologically important for other reasons. It might also lead to neglecting many other important features of the natural world. In recent years there have been great declines in the abundance of common species across many ecological communities (Gaston 2010, 2011). Such declines could in principle *increase* measures of heterogeneity, while representing major ecological losses overall. Declines in abundance are matters of potential conservation concern, and might be obscured by focusing on diversity.

Many reasons for conservation have little to do with biodiversity as we have defined it. Forests may be valued as homes to particularly charismatic or culturally important plants and animals, or as a source of key natural resources. These conservation reasons arise from human relationships to particular animals, plants, and communities. Another important ground for conservation, again unrelated to diversity, has to do with moral duties towards individual living things. Rawles (2004), for instance, has argued that the primary reason to be concerned with the destruction of ecosystems is grounded in respect for the lives and ends of individual animals, rather than patterns of difference or similarity between living things. And Maier (2012) argues that there is a particular value in relating the natural world through a principle of ‘leaving it alone’—a value that he sees threatened by ideas of biodiversity as something to be maximized and managed. These arguments suggest that the value associated with ecosystems and living communities is not exhausted by how varied or heterogeneous they are.

### A pluralist approach to ecological values

We therefore agree that protection of biodiversity should not be the sole aim of conservationists. However, it does not follow that we should eliminate protection of biodiversity as an aim of conservation biology. Instead, it might show that we should adopt a pluralist approach, and treat biodiversity is one of many valuable features of the natural world. A pluralist might hold the following commitments:

We have reason to value many features of the natural world.

Biodiversity is one these features.

On this picture, the sheer variety of life is one of the things to be valued in the natural world, but it is not the only ground of value. It is no problem for this pluralist view that biodiversity cannot capture all of the values associated with ecosystems, since there are other objects of value apart from biodiversity. We have already given a good reason for seeing the sheer variety of life as something to be valued. This reason is expressed in what Darwin describes as his sense of wonder at nature’s ‘endless forms most beautiful’.

Santana’s argument hinges on the idea that biodiversity is acting as a placeholder, meant to stand for whatever it is that we value about ecological communities and the natural world (2014, p. 765). However, we need not think of biodiversity as a placeholder for ‘biological value’. Instead, we may think that biodiversity is one of many features of the natural world to be valued in and of itself. If biodiversity is valuable, then conservationists have reason to protect it, even if they also have reason to protect other features of the natural world.

Consider an analogous position in the philosophy of wellbeing. Objective list theorists claim that a plurality of basic goods are constituents or components of well-being. Guy Fletcher, for example, claims that all of the following goods are components of wellbeing: “Achievement, Friendship, Happiness, Pleasure, Self-Respect, Virtue,” (2013, p. 214). Now imagine someone objecting that we should not aim to promote self-respect, because self-respect does not capture everything that is important for wellbeing. This objection is structurally similar to Santana’s, but it is not a strong argument against the value of self-respect. Self-respect does not capture all aspects of wellbeing, but this does not imply that self-respect is not valuable. A person who lacked self-respect would be missing something important, even if she had an abundance of the other goods associated with wellbeing.

In the same way, it could be argued that a world in which a great variety of living things had existed but in which most of this variety of forms of life had died off, would thereby have lost something valuable, even if there were many other good things about it. On this view, the diversity of life is a constitutive part of the good of ecological communities, even if there are many other features that are also good.

It might be objected that biodiversity is not really valuable, because a commitment to maximizing biodiversity would lead to absurd implications. Maier (2012) expresses concern about ideals of maximizing diversity, and Santana uses hypothetical maximizing as a test for biodiversity’s value. He suggests that if we really valued the diversity of life as such, then that would mean we should try to maximize it (2014, p. 769). Maximizing biological diversity might mean genetically engineering as many new species and forms of life as possible, or introducing as many new species of fish into a lake as possible. It is not obvious that we should do either of these things. Therefore, biodiversity must not really be valuable.

However, this objection does not undermine the claim that biodiversity is valuable. Instead, it illustrates the limitations of assuming that practical values should be maximized. Some philosophical theories hold that ultimate value is to be maximized. For instance, many consequentialists hold that *the good* is to be maximized. But it cannot be reasonably maintained that we should maximize practical values. By practical values, we mean the kinds of things that feature in practical normative reasoning as the objects of positive valuing attitudes. In the domain of practical values, reasoning which proceeds by maximizing is often inadequate and would lead to unpalatable conclusions.

For instance, friendships are held to be of great value, but this does not mean that we should maximize the number of friends we have (or the intensity of friendships, etc.). Nor does it mean that all friendships are good or that friendship is valuable under all circumstances. Instead, as with all practical values, friendships are defeasibly good, to be enjoyed in balance with other good things. Likewise, a rocky cliff may be aesthetically valuable, but it would be wrong to interpret this as a claim that rocky cliffs should be ‘maximized’ in some way, for instance that we should maximize the number and quality of rocky cliffs. Thus it is no objection to our view that adopting a principle of maximizing biodiversity would lead to absurd outcomes. To claim that biological diversity is valuable is not to claim that it should be maximized, but that it should be respected, appreciated and protected in balance with other features that call for appreciation and protection.^9^

A second worry has to do with instrumental versus non-instrumental values. By describing biodiversity as valuable ‘in itself’, we have implied that biodiversity is important because it is non-instrumentally valuable. According to the standard view in value theory, something is instrumentally valuable if it is valuable as a means to obtaining something else that is valuable, whereas it is non-instrumentally valuable if it is valuable not merely as a means but in and of itself.^10^ By saying that biodiversity is non-instrumentally valuable, we mean that it is valuable as an end in itself and not merely as a means to obtain something else that is valuable. It might be objected that the most important reasons for attributing value to biodiversity are instrumental, instead of non-instrumental. The non-instrumental value of biodiversity is not universally agreed upon, and reflects cultural preferences. By contrast, it might be argued that biodiversity has many instrumental benefits, as least some of which may be relatively uncontroversial at this stage, for instance as a source of pharmaceutical innovation, and perhaps as a supporter of ecosystem functions such as productivity and robustness.

However, this does not constitute an objection to our view. For one thing, one might think that biodiversity’s value is only instrumental, but that a) its benefits are very important, and b) nothing else can replace biodiversity in serving these instrumental purposes. In that case, it is instrumentally valuable, but not easily substitutable, and so we should take care to protect it. Thus we might conclude that biodiversity is one of many valuable features of the natural world and should be protected carefully in and of itself, even if biodiversity’s value is only instrumental.

Moreover, there is no contradiction in thinking that biodiversity possesses both instrumental *and* non-instrumental value, and that we have reasons related to both of these. Just as an objective list theorist can hold that friendship is good for its own sake as well as for the pleasure it brings, so a conservation biologist can hold that biodiversity is valuable for its own sake and for the ecosystem services that it provides. We can, then, accept that biodiversity has significant instrumental value while holding that it is also non-instrumentally valuable. Our own view is that biodiversity has both instrumental and non-instrumental value.

Santana does not seriously consider the possibility that biodiversity is non-instrumentally valuable—that biological diversity is something to be valued for its own sake, not as a means to a further end. Insofar as biological diversity itself is valuable, he takes its value to be instrumental, and then claims that since its instrumental value is not unambiguous, it is better not to treat it as valuable at all. There is one short passage in which Santana acknowledges that we might, following Sober (1986), hold that biodiversity is of non-instrumental aesthetic value. However, he then claims that even on this view biodiversity must be ‘only an instrumental end aiming ultimately at aesthetic value’ (2014, p. 774). He makes the further assumption that if biodiversity is an object of aesthetic value, then it must be fully substitutable with any other such object, and thus is not distinctively important in itself.

There are several problems with this argument. First, Santana’s argument against the instrumental value of biodiversity is problematic. According to leading ecologists, current evidence provides good reason to believe that biodiversity *is* associated with ecosystem robustness and other aspects of ecosystem well-functioning (Balvanera et al. 2006; Cardinale et al. 2012; Hooper et al. 2005), so Santana’s summary could be seen as downplaying the extent of current scientific agreement on the instrumental importance of biodiversity. In light of existing evidence, the fact that we do not yet have a clear understanding of the precise relationships between biodiversity and other ecosystem functions speaks in favour of a precautionary approach. It is difficult to recover diversity once it is lost, and diversity seems to have some important and complex relationships to other ecosystem functions, but we do not yet know very much about these relationships. This suggests that we should protect it while we learn more.

Moreover, it is mistaken to say that appreciating biodiversity aesthetically amounts to viewing it instrumentally, as a mere means (Brady 2003, p. 34). As Hume and Kant both emphasise, there is a qualitative difference between instrumentally valuing something as a mere means, and valuing something as an end, with appreciative attitudes directed towards the object itself. (Hume 2006, pp. 90–96; on Kant, see Brady 2003, p. 34). To say that biodiversity is non-instrumentally valuable is to say that it warrants our appreciation, in and of itself, and not as a mere means to other ends.^11^

It is ethically significant that many people do value biodiversity in a non-instrumental way. Popular scientist David Quammen connects biodiversity loss to a wide range of non-instrumental human values, writing ‘Within a few decades, if present trends continue, we’ll be losing a lot of everything. As we extinguish a large portion of the planet’s biological diversity, we will lose also a large portion of our world’s beauty, complexity, intellectual interest, spiritual depth, and ecological health’ (1996, p. 607). This value was reflected earlier in Wallace’s description of his ‘intense interest in the mere variety of living things’, which he describes as a ‘wonderful variety in nature—this overwhelming, and, at first sight, purposeless wealth of specific forms among the very humblest forms of life’ (quoted in Berry 2008). Not everybody shares these attitudes, making the value of biodiversity more contested than the value of, say, happiness. Nevertheless, recognizing that many people value biodiversity in this way gives us *pro tanto* reasons to respect and protect this feature of the natural world.

Santana claims that even if biodiversity is non-instrumentally valuable, this is of little importance for conservation decision-making, on the grounds that ‘Our limited resources for conservation demand that we prioritize some units over others, so if all units are equally intrinsically valuable, recognizing intrinsic value fails to help us to make comparative decisions’ (2014, p. 774). Similar concerns are expressed in Maclaurin and Sterelny (2008) and Justus et al. (2009b). However, holding that biodiversity is valuable intrinsically is not the same as holding that all components are equally valuable, or that they must be protected at all costs. Practical reasoning often requires us to make comparative judgments between things that we value intrinsically, or non-instrumentally. For instance, we might value achievement for its own sake, but think that it is not worth sacrificing self-respect or friendship to gain it.

Of course, establishing that friendship and achievement are intrinsically valuable is not sufficient on its own to guide action; for that, we need to develop an understanding of how to balance these different values. Moreover, there might be cases where it seems impossible to make a comparative decision about which value is most important. However, it does not follow from this that we have *no reason* to promote friendship, achievement or pleasure. Instead, it shows that doing so requires further normative judgment. Analogously, the mere claim that biodiversity is intrinsically valuable does not by itself tell us how biodiversity should be weighed against other sources of value.^12^ Balancing competing values in conservation requires judgment. However, this does not mean that good comparative judgments are impossible. Even if there are some cases where there is no determinate answer about which strategy to choose, much of the time there will still be better and worse choices. For instance, ‘biodiversity hotspots’ provide one tool for supporting such decisions. Biodiversity hotspots are regions of high biodiversity that are facing intense environmental pressures. At least 50% of vascular plant species and 42% of terrestrial vertebrates (amphibians, mammals, birds, and reptiles) are contained within 34 biodiversity hotspots, covering 3.4 million km^2^, or 2.3% of the world’s land mass (Mittermeier et al. 2011). Thus much of the world’s species diversity is thought to be concentrated in a few dozen geographical regions. Conservationists have good reason to protect these habitats. Nevertheless, many conservation decisions will not be so straightforward. This may be disappointing, as it may have been hoped that defining biodiversity well would provide us with a rubric for conservation decision-making. However, in our view biodiversity is not the kind of concept that can simply settle our conservation decisions. Instead, judgment will be required to determined which dimensions of diversity are most important in a given context, and to understand how to balance these with other conservation concerns.

Finally, even if it is worth protecting many features of ecosystems, it might still be advantageous to treat biodiversity as a primary target of conservation. Policies aimed at protecting biodiversity often have wider benefits. Habitat loss is the single greatest threat to biodiversity. However, habitat loss also threatens abundance, productivity, nutrient cycling, and other aspects of ecosystem functioning. Protecting biodiverse habitat may be a relatively straightforward policy goal, and a good way of protecting a wide range of goods. For instance, research in Southeast Asian found that areas protected by biodiversity conservation policy had better outcomes in terms of poverty than similar areas that were not protected (Turner et al. 2012). Thus it is possible to identify synergies between policy goals, even if the aims are distinct. While not decisive, these arguments suggest that even ecological pluralists may in many cases still have good practical reason to treat biodiversity as a primary conservation target.

In this section, we have defended the normative role of biodiversity against Santana’s challenge. We have argued that although biodiversity does not capture all objects of conservation concern, it is one non-instrumentally valuable feature of the natural world. However, it might be objected that this response is too weak to safeguard the central role that biodiversity currently plays in conservation decisions.^13^ Biodiversity is held to be valuable enough to warrant the creation of major international treaties to safeguard its protection. All we have said is that biodiversity is one valuable feature of the natural world among others. This might not seem sufficient to show that biodiversity should continue to the central role in conservation planning and decision making that it currently occupies.

We offer two responses to this objection. First, it should be noted that our aim in this paper has not been to provide a full account of the extent to which biodiversity should influence conservation decision-making. We have rather sought to show that biodiversity can have a role in conservation, even if we accept that it does not capture all objects of conservation concern. Second, given that many people take biodiversity to be an important element of conservation decision-making, worthy of international treaties, there seems to be a *prima facie* case for thinking that it is sufficiently valuable to warrant a role in conservation. Santana’s challenge can be seen as an attempt to undermine this *prima facie* case. We have shown that Santana’s argument against this *prima facie* case can be dismissed. While far from a full defence of the central role that biodiversity plays in conservation decision-making, this places the burden of proof on those who think we should eliminate the concept of biodiversity from science and environmental ethics.

## Conclusion

On a classic multidimensional conception, biodiversity refers broadly to the variety of life. Since living things can be compared along innumerable dimensions, biodiversity is fundamentally multi-dimensional, and which dimensions are most important will depend upon explanatory aims. In this paper, we have defended the importance of this classic multidimensional conception of biodiversity.

One challenge for defenders of biodiversity is to show that the concept itself is useful and coherent. Eliminativists like Carlos Santana have argued that we should do away with the umbrella concept of biodiversity, on the grounds that different dimensions of biodiversity are only loosely correlated. No dimension can serve as an adequate surrogate for the rest, or as a measure of overall biodiversity. Since scientists cannot measure overall biodiversity, he argues, they should eliminate the umbrella concept, and restrict themselves to naming the more specific dimensions being measured.

However, this does not show that the concept should be eliminated. The fact that biodiversity cannot be described as a simple magnitude does not undermine its importance. There is no need for the dimensions of biodiversity to covary in order for it to play important roles within science as both explanandum (a phenomenon of scientific interest, to be described and explained) and explanans (a property featuring in an explanation). Instead, there is just a need for the concept to help systematize understanding, for instance by revealing the unity in distinct phenomena.

A second challenge for defenders of a classic multidimensional conception of biodiversity is to articulate its normative importance. What role should biodiversity play in conservation efforts? It seems clear that protecting diversity should not be the sole aim of conservationists. Many valuable features of ecosystems are not readily characterised in terms of diversity. However, just because biodiversity does not capture all objects of conservation concern does not mean that we should abandon it as a target of conservation efforts. The sheer variety of life is something to be valued in and of itself. If biodiversity is valuable, then conservationists have reason to protect it, even if they also have reason to protect other features of the natural world. Indeed, in many cases protecting biodiversity may have the effect of protecting a wide range of other values as well.

## Footnotes

1 For instance, in a major work on biodiversity and biogeography, Stephen P. Hubbell defines biodiversity as ‘synonymous with species richness and relative species abundance in space and time', and proposes that this focus on species is ‘in keeping with the classical discipline of ecology as the scientific study of the distribution and abundance of species and their causes' (Hubbell 2001, p. 3).

2 Thanks to Alexander Bird, James Justus, Samir Okasha, and Kit Patrick for helpful discussion of this section.

3 The next two sections (“Two alternatives”, The conceptual importance of biodiversity sections) focus on scientific issues related to the conceptualization of biodiversity. Readers who are primarily interested in the *value* of biodiversity and other normative and ethical issues may prefer to skip to “The normative importance of biodiversity” section.

4 Further development of this work can be found in Lean and Maclaurin (2016) and Lean and Sterelny (2017).

5 This conclusion is reaffirmed in Lean and Maclaurin (2016) and Lean and Sterelny (2017).

6 Note, therefore, that we reject what Maier calls the ‘biodiversity project’, which attempts to capture all of the value of the natural world through the framework of biodiversity.

7 This view is neutral with regards to whether higher-level properties like diversity can have causal effects, or whether it is only the particular, micro-level instantiations that are causal. In either case, higher-level properties may play a valuable role in scientific explanation.

8 For a paper that focuses on explaining variation in effects instead of general trends.

9 Nothing here speaks against consequentialism, although it speaks against a certain kind of consequentialist reasoning in the practical domain. Consequentialists can simply say that maximizing the good means responding in appropriate ways to practical values like friendship, and that the theory offers a criterion of rightness but not a decision procedure. For a defence of this form of consequentialism see Railton (1984).

10 For important treatments of the distinction between instrumental and non-instrumental value in environmental ethics and aesthetics, see O’Neill (1992) and Brady (2003). A general statement of what we take to be the standard view of instrumental value is given in Schroeder (2016). This contrasts with some uses of the term ‘instrumental value’ that can be found in the literature. For example, Justus et al. define instrumental value as: “value that depends on valuers,” (2009a, p. 189). We take this definition, though, to better fit with what John O’Neill (1992, p. 120) labels subjective value, which is value arising from our valuing attitudes. This is to be contrasted with objective value which O’Neill defines as, “value an object possesses independently of the valuations of valuers,” (1992, p. 120). As O’Neill points outs, the term intrinsic value is used ambiguously between non-instrumental value, objective value, and non-relational value. In our discussion, we will use intrinsic value to mean non-instrumental value, i.e. valuable as an end and not merely as a means.

11 As noted earlier, we take this to be the standard view in value theory, but this treatment of the instrumental/non-instrumental value distinction does differ from some that can be found in the literature. On the account of instrumental value given by Justus et al. (2009a, b) even if we are valuing something as an end in itself it may still count as instrumental value.

12 Indeed, it has been argued by Justus et al. (2009b) that intrinsic values are incapable of informing conservation decision making. For a discussion of this argument see Sagoff (2009) and Justus et al. (2009b).

13 Thanks to an anonymous referee for raising this concern.
